# **Influencing factors affecting health-promoting lifestyles in patients with primary liver cancer: a latent profile analysis**

**DOI:** 10.1038/s41598-025-02987-9

**Published:** 2025-05-27

**Authors:** Xuerui Wang, Jiarong Ding, Liping Zhou, Qiaomei Fu, Ye Zhang, Yao Lu

**Affiliations:** 1 Division of Hepatobiliary and Transplantation Surgery, Department of General Surgery, Nanjing Drum Tower Hospital, (The Affiliated Hospital of Nanjing University Medical School), Nanjing, Jiangsu China; 2 Department of Urology Surgery, Nanjing Drum Tower Hospital, (The Affiliated Hospital of Nanjing University Medical School), Nanjing, Jiangsu China; 3 Department of Surgery, Nanjing Drum Tower Hospital, (The Affiliated Hospital of Nanjing University Medical School), Nanjing, Jiangsu China

**Keywords:** Primary liver cancer, Health promotes lifestyle, Health literacy, Potential profile analysis, Analysis of influencing factors, Cancer, Oncology

## Abstract

To explore the potential profile categories of health promoting lifestyle in patients with primary liver cancer and analyze the influencing factors of different categories. Primary liver cancer patients who visited a tertiary hospital in Nanjing from October 2021 to May 2023 were selected as the survey subjects. A general information questionnaire and a health-promotion lifestyle scalewere used for data collection. Potential profile analysis was conducted to classify the health-promoting lifestyles of patients with primary liver cancer, and the influencing factors of different categories were explored through univariate analysis and multiple logistic regression analysis. Clinical registration trial number: ChiCTR2400079823 (12/01/2024). A total of 328 questionnaires were collected, of which 324 were valid, resulting in a validity rate of 98.78%.The latent profile analysis indicates that the health promotion lifestyle of primary liver cancer patients can be categorized into three potential profiles: “low health promotion neglect” (*n* = 128, 39.5%), “moderate health promotion balance” (*n* = 87, 26.9%), and “high health promotion lone hero” (*n* = 109, 33.6%). The influencing factors include educational level, per capita monthly income, disease-related economic burden, postoperative duration, work status, sleep disorders, and whether neoadjuvant therapy was administered preoperatively (*P* < 0.05). Conclusion: There is significant heterogeneity in the health promotion lifestyles of patients with primary liver cancer. Nursing staff should develop targeted health promotion strategies, focusing on improving patients’ health behaviors to enhance their quality of life.

Primary liver cancer (PLC) is one of the most common malignant tumors in China, ranking as the sixth largest cancer and the third leading cause of cancer-related deaths worldwide^1^. The number of PLC deaths and new cases in China accounts for about half of the global total, and the incidence rate and mortality rate is about twice the world average^2^. With the advancement of comprehensive treatment technology for liver cancer and the improvement of perioperative management, the prognosis of patients has been improved, and the 5-year survival rate has increased. Researches have shown^3,4^ that the occurrence of liver cancer is not only related to genetic factors and past illnesses, but also closely related to unhealthy lifestyles such as unhealthy diet, lack of physical exercise, and smoking and alcohol abuse. A 2022 Lancet study found that a healthy lifestyle has a cumulative protective effect on liver cancer^5^, with participants who adhere to 4 or 5 healthy lifestyle factors having a 43% lower risk of liver cancer compared to those who only adhere to 0 or 1 healthy lifestyle factor.

Patients with primary liver cancer (PLC) face multiple physiological and psychological burdens, significantly affecting their health behaviors. These include physiological limitations, side effects of treatment, and restrictions in psychosocial functioning. Due to impaired liver function, PLC patients experience reduced metabolic capacity, leading to fatigue, malnutrition, and drug metabolism disorders, which limit exercise compliance and dietary adjustments^6,7^. Postoperative complications, such as bile leakage and infections, further weaken their self-management capabilities. Gastrointestinal reactions induced by chemotherapy and targeted therapies, such as nausea and diarrhea, may exacerbate irregular eating patterns^8^. Immunotherapy-related fatigue syndrome can decrease patients’ willingness to exercise^9^. Additionally, the stigma associated with a cancer diagnosis, anxiety about recurrence, and economic pressures result in depression or anxiety symptoms in 40-60% of patients, leading to avoidance of health consultations and social support. Overprotection by family members or communication barriers may suppress patients’ autonomy in adopting health behaviors.

Health promoting lifestyle refers to all activities taken by individuals to maintain or promote health, achieve self realization or satisfaction. Improving the level of health promotion lifestyle is beneficial for expanding patients’ health potential, improving disease control, thereby delaying complications, and improving quality of life. However, there is currently little attention paid to the health promotion lifestyle of PLC patients. The existing studies mainly conduct cross-sectional analysis based on the total score of the scale^10,11^, ignoring individual heterogeneity and failing to identify the behavioral characteristics differences of patient subgroups. In addition, the existing interventions are mostly based on a unified health education curriculum, and lack of hierarchical strategies designed for different behavioral characteristics of people, resulting in the accuracy and pertinence of health promotion strategies for PLC patients being limited. Latent Profile Analysis (LPA)^12^ is a method that focuses on individuals to determine their potential feature classification and further study the characteristics of different potential categories of populations. Therefore, this study used LPA to explore the characteristics and influencing factors of different categories of health promoting lifestyles in PLC patients, aiming to provide reference for clinical workers to develop targeted intervention strategies.

## Data and methods

### Survey subjects

Select primary liver cancer patients admitted to the Hepatobiliary Surgery Department of a tertiary hospital in Nanjing from October 2021 to May 2023 as the survey subjects. Inclusion criteria: Patients diagnosed with primary liver cancer according to the “Diagnosis and Treatment Standards for Primary Liver Cancer (2019 Edition)”^13^; Individuals aged 18 to 85 years old; Patients who have undergone liver tumor resection surgery within the past year; The postoperative general condition is good, with normal communication and understanding abilities; Informed consent and voluntary participation in this study. Exclusion criteria: those who cannot take care of themselves or have unstable health status (such as Barthel index < 60 points or vital signs not reaching the stability standard, and have serious laboratory abnormal indicators (such as albumin < 25 g/l, platelet < 50 × 10^9^/L, etc.); With serious complications (such as liver failure, active bleeding, abdominal infection, biliary fistula, etc.); Those with neurological or psychiatric disorders or communication disorders; Critically ill patients. The sample size was determined through a dual approach: For the unordered multiclass logistic regression with 25 independent variables, we applied the rule of 10 events per variable (EPV)^14^, yielding 25 × 10 = 250 cases. To accommodate an estimated 20% dropout rate, the adjusted target was calculated as 250/(1 − 0.2) = 313, rounded up to 320. For latent profile analysis (LPA), a minimum of 200 cases is recommended to ensure model stability. The larger value (320) was selected as the target. Ultimately, 324 participants were enrolled after excluding incomplete responses. This study has been approved by the hospital ethics committee (2019-094-01). Clinical registration trial number: ChiCTR2400079823(12/01/2024).

### Investigation tools

#### General information survey form

Designed based on literature review by researchers and consultation with clinical nursing experts, including (1) general demographic characteristics and disease-related data General demographic characteristics include patient age, gender, marital status, educational level, family residence, work status, per capita monthly income, and whether there is medical insurance; (2) disease characteristics include the duration of disease diagnosis, surgical method, postoperative duration, body mass index, level of understanding of the disease, economic burden caused by the disease, participation in disease treatment selection, presence of chronic diseases, sleep disorders, preoperative neoadjuvant therapy, and postoperative adjuvant therapy.

#### Health promotion lifestyle scale

The Health Promoting Lifestyle Profile (HPLP) was developed by Walker et al.^15^. in 1987 and consists of 48 items. This study used the revised version of HPLP, HPLP-II^16^, which has been applied in populations such as cancer patients, community residents, adolescents, and the elderly^17^ in China, with the aim of evaluating individual health promoting lifestyles. HPLP-II consists of 52 items, including six dimensions: spiritual growth, health responsibility, sports, nutrition, stress management, and interpersonal relationships. Using a Likert 4-point rating system, scores 1–4 represent “never”, “sometimes”, “often”, and “always”, with a total score range of 52–208. The higher the score, the better the health behavior. Scores 52–89 indicate poor health promoting lifestyle, 90–126 indicate average, 127–163 indicate good, and 164–208 indicate excellent. The Cronbach’s alpha coefficient of the scale is 0.943, and the test-retest reliability coefficient is 0.892^18^. The Cronbach’s alpha coefficient of the scale in this study is 0.976.

### Data collection and quality control methods

Before conducting a formal survey, select 3–5 research subjects in advance to conduct a pre survey, determine the time required to fill out the questionnaire, and provide guidance for the investigator to explain the purpose, significance, and meaning of the items to ensure the quality of the survey. The formal questionnaires were interviewer-administered by trained research assistants. Prior to data collection, all investigators completed a 4-hour standardized training program covering questionnaire content (e.g., definitions of key terms), neutral communication techniques, and data entry protocols. Role-playing sessions were conducted to ensure consistent interpretation of sensitive items (e.g., mental health questions). The disease characteristics are obtained by researchers after reviewing medical records. For those who are limited by their educational level or visual acuity, the investigator will read the questions one by one for patients to choose from. The questionnaire will be collected on site and checked for completion status. If there are any omissions, they will be supplemented on the spot. If there are regular responses or all options are the same, the questionnaire will be deemed invalid.

### Statistical methods

All data is entered and checked by two people. Use Mplus 8.3 and SPSS 26.0 software for data analysis. The potential profile model is used to fit indicators^6^, including AIC, BIC, aBIC, Entropy, LMR, and BLRT based on Bootstrap for evaluation. Use Harman’s single factor test to perform a common method bias test on scale items^19^. Quantitative data that conforms to normal distribution are expressed as mean and standard deviation, and one-way ANOVA is used for multi group comparison; Quantitative data are presented in terms of examples and percentages (rates), and multiple group comparisons are conducted using chi square test or Kruskal Wallis rank sum test with multiple independent samples. Adopting unordered multiclass logistic regression analysis to investigate the potential influencing factors of health promoting lifestyle categories in patients with primary liver cancer. *P* < 0.05 indicates statistical significance of the difference.

## Results

### Univariate analysis of general information and potential categories of health promoting lifestyle in patients with primary liver cancer

A total of 328 questionnaires were distributed and collected in this study. After excluding 4 invalid questionnaires, 324 valid questionnaires were collected, with a valid questionnaire collection rate of 98.78%. The HPLP-II score of 324 patients with primary liver cancer was (124.53 ± 23.08) points, of which 9 patients (2.8%) scored below 89 points, 197 patients (60.8%) scored between 90 and 126 points, 106 patients (32.7%) scored between 127 and 163 points, and 12 patients (3.7%) scored above 164 points. Its general information is shown in Table 1. The results of univariate analysis showed that there were statistically significant differences (*P* < 0.05) among the three categories of patients in terms of education level, work status, per capita monthly income, disease-related economic burden, postoperative duration, understanding of the disease, choice of disease treatment methods, sleep disorders, and whether they received neoadjuvant therapy before surgery.

**Table 1:** General data and univariate analysis of potential categories of health promoting lifestyle in patients with primary liver cancer [cases(percentages, %)]

Item	Cases	C1(*n* = 128)	C2(*n* = 87)	C3(*n* = 109)	C^2^ / H	*P*
	(*n* = 324)					
Age(years)					1.537^b^	0.464
≤ 45	57(17.6)	27(21.1)	12(13.8)	18(16.5)		
46 ~ 60	132(40.7)	51(39.8)	36(41.4)	45(41.3)		
≥ 61	135(41.7)	50(39.1)	39(44.8)	46(42.2)		
Sex					1.550^a^	0.461
Male	218(67.3)	85(66.4)	63(72.4)	70(64.2)		
Female	106(32.7)	43(33.6)	24(27.6)	39(35.8)		
Marriage					0.008^a^	0.996
Married	306(94.4)	121(94.5)	82(94.3)	103(94.5)		
Single	18(5.6)	7(5.5)	5(5.7)	6(5.5)		
Educational background					32.696^b^	<0.001
Illiteracy	93(28.7)	48(37.5)	29(33.3)	16(14.7)		
Junior high school and below	91(28.1)	43(33.6)	22(25.3)	26(23.9)		
High school or vocational school	79(24.4)	28(21.9)	20(23.0)	31(28.4)		
College degree or above	61(18.8)	9(7.0)	16(18.4)	36(33.0)		
Residence					2.798^a^	0.247
Citiea and Towns	167(51.5)	73(57.0)	40(46.0)	54(49.5)		
Rural	157(48.5)	55(43.0)	47(54.0)	55(50.5)		
Work status					41.435^a^	<0.001
On the job	125(38.6)	25(19.5)	35(40.2)	65(59.6)		
No job	138(42.6)	73(57.0)	38(43.7)	27(24.8)		
Quit	61(18.8)	30(23.4)	14(16.1)	17(15.6)		
Per capita monthly income					8.346^b^	0.015
>5000	158(48.8)	53(41.4)	41(47.1)	64(58.7)		
3000 ~ 5000	85(26.6)	35(27.3)	26(29.9)	24(22.0)		
<3000	79(24.4)	40(31.3)	20(23.0)	19(17.4)		
Do you have medical insurance					7.400^a^	0.116
Yes	273(84.3)	115(89.8)	69(79.3)	89(81.7)		
No	50(15.4)	13(10.2)	17(19.5)	20(18.3)		
Degree of disease-related economic burden					9.631^b^	0.008
Heavy	106(32.7)	58(45.3)	24(27.6)	24(22.0)		
Fair enough	129(39.8)	39(30.5)	38(43.7)	52(47.7)		
No pressure	89(27.5)	31(24.2)	25(28.7)	33(30.3)		
Diagnosis duration					3.034^b^	0.219
≤ 1 year	158(48.8)	70(54.7)	40(46.0)	48(44.0)		
More than 1 year	166(51.2)	58(45.3)	47(54.0)	61(56.0)		
Surgical method					2.714^a^	0.607
Open	56(17.3)	27(21.1)	11(12.6)	28(25.7)		
Endoscope	133(41.0)	51(39.8)	37(42.5)	45(41.3)		
Robot	135(41.7)	50(39.1)	39(44.8)	46(42.2)		
Postoperative duration					32.408^b^	<0.001
< 1 month	98(30.2)	51(39.8)	27(31.0)	20(18.3)		
1–3 months	89(33.6)	43(33.6)	26(29.9)	20(18.3)		
3–6 months	77(23.8)	21(16.4)	23(26.4)	33(30.3)		
6 months to 1 year	60(18.5)	13(10.2)	11(12.6)	36(33.0)		
BMI					0.034^a^	0.983
Normal	150(46.3)	59(46.1)	41(47.1)	50(45.9)		
Obesity or thinness	174(53.7)	69(53.9)	46(52.9)	59(54.1)		
The level of understanding of the disease					7.703^b^	0.021
Very familiar	90(27.8)	28(21.9)	19(21.8)	43(39.4)		
Commonly	120(37.0)	51(39.8)	35(40.2)	34(31.2)		
Do not understand	114(35.2)	49(38.3)	33(37.9)	32(29.4)		
Whether to participate in the selection of disease treatment methods					6.355^a^	0.042
Yes	149(46.0)	69(53.9)	39(44.8)	41(37.6)		
No	175(54.0)	59(46.1)	48(55.2)	68(62.4)		
Combined chronic diseases					3.252^a^	0.197
Yes	221(68.2)	83(64.8)	66(75.9)	72(66.1)		
No	103(31.8)	45(35.2)	21(24.1)	37(33.9)		
Sleep disorders					6.532^a^	0.038
Yes	169(52.2)	73(57.0)	50(57.5)	46(42.2)		
No	155(47.8)	55(43.0)	37(42.5)	63(57.8)		
Did you receive neoadjuvant therapy before surgery					19.006^a^	<0.001
Yes	162(50.0)	54(42.2)	35(40.2)	73(67.0)		
No	162(50.0)	74(57.8)	52(59.8)	36(33.0)		
Whether to receive adjuvant therapy after surgery					0.339^a^	0.844
Yes	171(52.8)	65(50.8)	47(54.0)	59(54.1)		
No	153(47.2)	63(49.2)	40(46.0)	50(45.9)		

^a^*Χ*^*2*^; ^b^H.

### Common method deviation test results

The results showed that the eigenvalues of 33 factors were > 1, and the variance contribution rate of the first factor was 18.30% (< 40% of the recommended standard), indicating that there was no significant common method bias.

### Potential profile analysis results of health promotion lifestyle in primary liver cancer patients

Using the six dimensions of the HPLP-II scale as explicit indicators, establish 1–5 latent category models in sequence, as shown in Table 2. As the number of categories increases, the values of AIC, BIC, and aBIC decrease continuously. The entropy values of categories 2, 3, 4, and 5 are all > 0.8, and the LMRT and BLRT of categories 2 and 3 are statistically significant (*P* < 0.05). Although the entropy value of category 3 is lower than that of category 2, there is a clear inflection point in its steep slope map, and the average probability of patients belonging to each category is above 90%, indicating that the optimal model results obtained from potential profile analysis are reliable and have high discriminative power. Considering the practical significance of the model, Model 3 was ultimately chosen as the best fitting model.

**Fig. 1:**
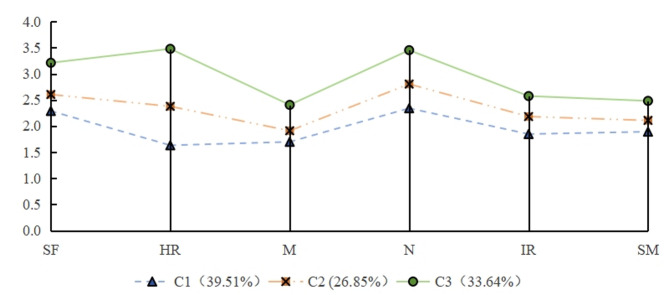
The characteristic distribution of 3 potential categories of the health promoting lifestyle among primary liver cancer patients.

Based on Model 3, the average scores of each category in the HPLP-II scale are shown in Fig. 1. The three categories are named according to the fluctuation of the dimensional score mean. The HPLP-II score of the first category (C1) is (101.66 ± 6.70) points, and patients in this category have the lowest scores in all dimensions, especially in the “health responsibility” dimension where the score is even lower. Therefore, it is named “Low Health Promotion-Neglectors”, accounting for 39.51% (128/324); The HPLP-II score of the second category (C2) is (121.83 ± 5.49) points, and the scores in all dimensions are at a moderate level, with relatively small fluctuations between dimensions. Therefore, it is named “Moderate Health Promotion-Balancers”, accounting for 26.85% (87/324); The HPLP-II score of the third category (C3) is (153.53 ± 6.48), with high scores in all dimensions, especially in “health responsibility”, where the score is the highest. However, “interpersonal relationships” and “stress management” are clearly at a lower level in the overall distribution, so it is named and “High Health Promotion-Lone Warriors”, accounting for 33.64% (109/324).

**Table 2:** Potential categories of health promoting lifestyle among primary liver cancer patients.

Model	AIC	BIC	aBIC	Entropy	*P*		Probability of classes
					LMR	BLRT	
1	2613.963	2659.332	2621.269	–	–	–	–
2	1145.324	1217.158	1156.892	0.976	<0.001	<0.001	0.664/0.336
3	691.127	789.427	706.957	0.967	<0.001	<0.001	0.399/0.336/0.265
4	623.903	748.668	643.995	0.933	0.5247	<0.001	0.255/0.400/0.323/0.021
5	567.767	718.996	592.12	0.898	0.2498	<0.001	0.398/0.009/0.321/0.256/0.015

### Multivariate analysis results of potential profile categories of health promotion lifestyle in patients with primary liver cancer

Using the potential profile categories of PLC patients’ health promotion lifestyle as the dependent variable, “low health promotion neglectful individuals”, “medium health promotion balanced individuals”, and “high health promotion lone heroes” were assigned values of 1, 2, and 3, respectively. The items with statistical significance in the univariate analysis were used as independent variables. Due to the P-value < 0.001 in the parallel test, unordered multiclass logistic regression was used for analysis, and the variables were screened out using a stepwise method. The assignment of independent variables is shown in Table 3. The results of logistic regression analysis showed that education level, per capita monthly income, disease-related economic burden, postoperative duration, work status, sleep disorders, and whether or not neoadjuvant therapy was the influencing factors of PLC patients’ health promoting lifestyle (*P* < 0.05), as shown in Tables 3 and 4.

**Table 3:** Assignment of independent variables.

Independent variable	Assignment method
Degree of education	Illiteracy (1,0,0), junior high school and below (0,1,0), high school or vocational school (0,0,1), college and above (0,0,0)
Work status	On duty (1,0), unemployed (0,1), resigned (0,0)
Per capita monthly income	>5000(1,0), 3000 ~ 5000(0,1), <3000(0,0)
Degree of disease-related economic burden	Heavy (1,0), decent (0,1), no pressure (0,0)
Postoperative duration	< 1 month (1,0,0), 1–3 months (0,1,0), 3–6 months (0,0,1), 6 months to 1 year (0,0,0)
The level of understanding of the disease	Very familiar with (1,0), generally (0,1), unclear about (0,0)
Whether to participate in the selection of disease treatment methods	Yes = 1, No = 0
Sleep disorders	Yes = 1, No = 0
Did you receive neoadjuvant therapy before surgery	Yes = 1, No = 0

**Table 4:** Multivariate logistic analysis results on influencing factors of latent profiles of health promoting lifestyle among primary liver cancer patients.

Item	C2				C3			
	β	*P*	OR	95%CI	β	*P*	OR	95%CI
Constant term	0.344	0.676			1.555	0.068		
Degree of education								
Illiterate	− 1.427	0.008	0.240	0.083 ~ 0.691	− 2.898	0.000	0.055	0.017 ~ 0.184
Junior high school and below	− 1.862	0.001	0.155	0.051 ~ 0.469	− 2.400	0.000	0.091	0.029 ~ 0.283
High school or vocational school	− 1.256	0.025	0.285	0.095 ~ 0.855	− 1.751	0.003	0.174	0.055 ~ 0.547
Work status								
On the job	1.293	0.007	3.645	1.426 ~ 9.318	1.493	0.003	4.448	1.656 ~ 11.946
Per capita monthly income								
>¥5000	0.788	0.050	2.199	1.000 ~ 4.835	1.290	0.005	3.632	1.462 ~ 9.024
Degree of disease-related economic burden								
Heavy	− 0.873	0.030	0.418	0.189 ~ 0.920	− 1.178	0.010	0.308	0.126 ~ 0.755
Postoperative duration								
<1month	− 0.433	0.415	0.648	0.229 ~ 1.839	− 1.371	0.011	0.254	0.089 ~ 0.728
1 ~ 3months	− 0.167	0.783	0.847	0.258 ~ 2.772	− 2.172	0.000	0.114	0.035 ~ 0.368
Sleep disorders								
No	− 0.022	0.961	0.978	0.399 ~ 2.395	1.289	0.006	3.631	1.436 ~ 9.178
Did you receive neoadjuvant therapy before surgery								
No	0.243	0.435	1.275	0.693 ~ 2.345	− 0.867	0.013	0.420	0.213 ~ 0.83

C1 is used as the reference group.

## Discussions

### There is significant heterogeneity in the level of health promotion and lifestyle among patients with primary liver cancer, which can be divided into three categories

The results of this study showed that the HPLP-II score of PLC patients was (124.53 ± 23.08) points, with “good” or “excellent” levels accounting for 36.42%, indicating that their health promoting lifestyle needs to be improved. This result is similar to the health promoting lifestyle survey conducted by Chen Nana et al.^12^ on patients with early gastrointestinal cancer, with a score of (125.94 ± 33.17). Possible negative impacts on patient health promotion may be related to the high malignancy of PLC, physical pain caused by complications, concerns about treatment efficacy, fear of cancer recurrence, and pressure on treatment costs^20^. The survey conducted by Zhao Xinhua et al.^21^ on the health promotion lifestyle of liver cancer patients with depressive symptoms [(98.12 ± 11.87) points] was significantly lower than the results of this study, indicating that negative psychology should be taken seriously in terms of its negative impact on patients’ health promotion lifestyle. The progress of systemic anti-tumor therapy and comprehensive therapy has brought new hope for improving surgical resection opportunities and treatment outcomes for patients with advanced liver cancer^20^. The health literacy and health management of PLC patients have received increasing attention. Therefore, medical staff should attach great importance to educating patients on healthy lifestyles, in order to enhance their ability to maintain and promote their own health and improve their quality of life.

This study identified significant heterogeneity in health promoting lifestyles of PLC patients. Based on statistical patterns of the data and clinical relevance of dimensional scores, three distinct profiles were ultimately derived: “Low Health Promotion-Neglectors”, “Moderate Health Promotion-Balancers”, and “High Health Promotion-Lone Warriors”. These classifications provide a foundation for exploring behavioral patterns in this unique population and designing stratified nursing interventions, with the aim of precisely improving health-promoting behaviors, optimizing health management systems, and ultimately enhancing quality of life. While these findings align with the health-promoting lifestyle typologies reported by Song et al.^22^ in breast cancer patients, PLC patients exhibit more specialized health management needs due to the aggressive nature of the disease and the complexity of treatment regimens. (a) In this study, the “Low Health Promotion - Neglectful” group accounted for 39.51% of the sample, representing the main category of health-promoting lifestyles. This indicates a need for focused screening and attention on the health-promoting lifestyles of these patients. The lowest scoring dimension, “Health Responsibility,” suggests a lack of health awareness among these patients, and their health behaviors such as health information acquisition, self-reporting of symptoms, health consultations, and regular follow-up visits need improvement. Therefore, nursing staff should assist these patients in establishing health management awareness. This can be achieved by utilizing the hospital follow-up system to create a “triggered health education” mechanism^23^. By identifying these patients through the electronic medical record system and sending customized health reminders postoperatively (such as follow-up schedules and symptom self-checklists), we can motivate them to actively learn and apply knowledge about PLC and health-promoting lifestyles, thereby improving health outcomes. (b) The “Moderate Health Promotion - Balancers” accounted for 26.85% of the sample. This group had a relatively small proportion and showed basic balance across dimensions, which aligns with the overall characteristics of the study’s participants. However, their HPLP-II levels were average and still have room for improvement. Nursing plans for these patients could include a needs assessment component, quantifying the priority of their needs (such as financial assistance and transportation convenience) through surveys, and providing stepped health education^4^. Collaborating with hospitals and social work departments, interventions can be tailored to provide “precision need matching.” (c) The “High Health Promotion - Lone Warriors” accounted for 33.64% of the sample. These patients had a strong health awareness and demonstrated high initiative and execution in managing their disease. However, they scored significantly lower in the “Interpersonal Relationships” and “Stress Management” dimensions, indicating challenges in interpersonal sensitivity, sociability, and stress regulation. Nursing staff need to focus on how these patients can achieve healthy relationships and adjust individual and even family stress. Future interventions could include constructing a “Stress-Social Support Dual-Channel” intervention: collaborating with psychology departments to design Mindfulness-Based Cognitive Therapy (MBCT) courses, and creating PLC patient mutual aid communities to alleviate stigma through peer support^16^. Family systems therapy could be introduced by inviting family members to participate in stress management workshops, improving communication patterns between patients and caregivers, and reducing family conflicts during the implementation of health behaviors.In the future, nursing staff can use psychological assessment tools to deeply explore the gap between the support patients expect and what they actually receive, and develop targeted intervention plans based on patients’ care needs.

### Potential categories of health promotion and lifestyle in patients with primary liver cancer are influenced by socioeconomic status

The results of this study showed that the characteristics of higher education level, economic income, working status (on-the-job), and lower evaluation of the economic burden caused by diseases constitute the high socioeconomic status attribute, and socioeconomic status has a significant positive predictive effect on the health promoting lifestyle of PLC patients, which is consistent with previous research^24^ and verifies the social causality theory. Socioeconomic status directly affects individuals’ access to health information and medical services. Therefore, maintaining the health equity of liver cancer patients, strengthening the popularization of liver cancer health science, and promoting patient participatory care models are of great significance in today’s increasingly valued health literacy among liver cancer survivors. In addition, occupational status is one of the key factors that influence individuals to adopt a health promoting lifestyle^25^. Research has shown that returning to work is beneficial for reducing the economic toxicity of cancer patients^26^, enhancing their social identity, personal achievement, and sense of social belonging^27^. However, the current situation of liver cancer patients returning to work is not ideal and faces many challenges^28^. This suggests that medical staff can increase their attention to the quality of life of liver cancer patients, take effective measures, develop work survival nursing plans, and help patients better return to work; On the other hand, special attention should be paid to the health awareness of non occupational PLC patients, starting from helping them establish good doctor-patient communication methods, obtaining disease-related knowledge and personal health status information, exploring active response resources, strengthening the significance of disease response, etc^29^. , strengthening their health responsibilities, guiding health behaviors, and promoting the improvement of health literacy.

### Potential categories of health promotion lifestyle for patients with primary liver cancer are influenced by the stage of disease treatment

The results of this study showed that PLC patients who did not receive neoadjuvant therapy within 3 months after surgery were more likely to be classified as “low health promotion neglectors”, which may be related to the interference of postoperative physiological trauma, physical discomfort, psychological pain, and other factors that led to a low state of health promotion behavior. Yang Xinyu et al.^30^ conducted a survey on 216 colorectal cancer survivors using HPLP-II and found that postoperative duration can positively predict health promoting lifestyle. The health promoting lifestyle scores of colorectal cancer survivors with less than 1 year were significantly lower than those with 1–3 years or more. It is suggested to pay attention to the level of health promotion for patients in the short term after surgery and strengthen nursing interventions. In addition, preoperative neoadjuvant therapy can help PLC patients accumulate symptom experience and coping experience during the disease treatment process, thereby promoting their HPLP level. With the continuous maturity of perioperative treatment concepts for liver cancer^31^, the connotation and extension of whole process management for liver cancer are undergoing changes. The health promotion lifestyle and health management issues of PLC patients are becoming increasingly prominent. Nursing staff can provide personalized nursing plans for patients with different treatment plans and stages. Wang Yanhui et al.^32^ included the “cycle and precautions of immunotherapy” in the discharge preparation plan for postoperative liver cancer patients, effectively enhancing their recovery confidence after discharge and improving the quality of discharge guidance. Zhang Lanju et al.^33^ developed a mobile nursing assistance application for PLC patient health literacy management, effectively improving patients’ health literacy level and enhancing their quality of life.

### Potential categories of health promoting lifestyle in patients with primary liver cancer are influenced by sleep quality

The results of this study indicate that PLC patients with sleep disorders have a lower level of health promoting lifestyle, which is consistent with the study by Zhong Qing et al.^34^. The team proposed that improving sleep behavior is a promoting factor in the formation of an overall healthy lifestyle by exploring the impact of sleep behavior on other health behaviors. Poor sleep health is often accompanied by more physical symptoms, psychological problems, and health issues, which are related to poorer quality of life, lower social functioning, and more medication use^35^. Further analysis shows that in the dimensions of HPLP-II in this study, the scores from low to high are as follows: exercise, stress management, interpersonal relationships, health responsibility, self actualization, and nutrition. It can be seen that PLC patients are particularly weak in terms of exercise, and exercise is widely considered a safe, economical, and easily accessible method to improve sleep^36^, which plays a significant role in coping with stress and promoting interpersonal relationships. Therefore, in nursing work, in addition to focusing on self-report of patient symptoms^10^, professional measurement tools such as the Sleep Health Index^37^, RU-SATED framework^38^, etc. can also be used for continuous and dynamic evaluation, and Just In Time Adaptive Intervention (JITAI) can be given^39^. In the future, further research can be conducted on the exercise intervention plan for PLC patients, and longitudinal studies can be combined to explore the timing when patients need or are most likely to accept stress management interventions. Personalized decision points, adaptive variables, and decision rules can be set up to actively or passively provide nursing interventions such as stress relief and emotional support.

## Limitations

This study is the first to use LPA to explore the health-promoting lifestyle of patients with different characteristics of primary liver cancer, therefore, more research is needed to support our findings. The study employed a cross-sectional design, which does not allow for the establishment of causal relationships between variables, and only discusses factors affecting the health-promoting lifestyle. Hence, future research can adopt a longitudinal study design for further investigation. This study has inherent limitations of a single-center design, including restricted geographic location and diversity of medical institutions, as well as potential selection bias due to non-random recruitment of subjects. We suggest that future researchers could adopt rigorously designed multi-center studies to address these limitations. We propose the following actionable framework: (1) Recruitment Strategy: Enroll patients from eastern (e.g., Shanghai), central (e.g., Wuhan), and western China (e.g., Chengdu) to capture socioeconomic diversity. Include tertiary hospitals (specialized oncology care) and community clinics (long-term management) at a 2:1 ratio to reflect real-world care pathways. (2) Data Standardization: Implement the REDCap electronic data capture system with predefined logic checks to ensure consistency. Conduct monthly cross-center audits using the CDISC SDTM standard, with discrepancies resolved via a steering committee. (3) Analytic Methods: Apply mixed-effects logistic regression to adjust for center-level clustering. Perform latent transition analysis (LTA) to track lifestyle profile changes longitudinally. These methods may help overcome the limitations of our study and assist in enhancing the generalizability of the research results and controlling methodological heterogeneity.

## Conclusion

There is significant heterogeneity in the health promotion lifestyle of PLC patients, with three categories identified: “low health promotion neglectful”, “moderate health promotion balanced”, and “high health promotion lone hero”. Research and development, education level, per capita monthly income, disease-related economic burden, postoperative duration, work status, sleep disorders, and whether preoperative neoadjuvant therapy affect the potential categories of PLC patients’ health promotion and lifestyle. Therefore, nursing staff should implement targeted intervention measures based on their classification characteristics. The survey subjects of this study were only from one hospital. In the future, the sample size can be expanded to conduct multi center, longitudinal studies to explore the characteristics of health promoting lifestyle in patients at different treatment stages and the trajectory of changes in health promoting lifestyle in PLC patients; Qualitative research can also be conducted to supplement quantitative research results, or empirical research can be conducted to verify causal relationships, providing stronger evidence support for the development of scientific and accurate nursing strategies.

## Data Availability

Due to concealment involving participants, privately anonymous datasets will be sent to by reasonable request corresponding author. If anyone wishes to obtain data from this study, they should contact Yao Lu.
